# Mismatch between shape changes and ecological shifts during the post-settlement growth of the surgeonfish, *Acanthurus triostegus*

**DOI:** 10.1186/1742-9994-9-8

**Published:** 2012-04-25

**Authors:** Bruno Frédérich, Orphal Colleye, Gilles Lepoint, David Lecchini

**Affiliations:** 1 Laboratoire de Morphologie Fonctionnelle et Evolutive, Institut de Chimie (B6c), Université de Liège, Liège, Belgium; 2 MARE, Laboratoire d’Océanologie, Institut de Chimie (B6c), Université de Liège, Liège, Belgium; 3 CRIOBE, USR 3278 – CNRS / EPHE, Centre de Recherches Insulaires et Observatoire de l’Environnement, Labex « CORAIL », CBETM – Université de Perpignan, Moorea, French Polynesia

**Keywords:** Acanthuridae, Allometry, Diet, Geometric morphometrics, Habitat change, Moorea Island, Reef fishes

## Abstract

**Background:**

Many coral reef fishes undergo habitat and diet shifts during ontogeny. However, studies focusing on the physiological and morphological adaptations that may prepare them for these transitions are relatively scarce. Here, we explored the body shape variation related to ontogenetic shifts in the ecology of the surgeonfish *Acanthurus triostegus* (Acanthuridae) from new settler to adult stages at Moorea Island (French Polynesia). Specifically, we tested the relationship between diet and habitat shifts and changes in overall body shape during the ontogeny of *A. triostegus* using a combination of geometric morphometric methods, stomach contents and stable isotope analysis.

**Results:**

After reef settlement, stable isotope composition of carbon and nitrogen revealed a change from a zooplanktivorous to a benthic algae diet. The large amount of algae (> 75% of stomach contents) found in the digestive tract of small juveniles (25–30 mm SL) suggested the diet shift is rapid. The post-settlement growth of *A. triostegus* is highly allometric. The allometric shape changes mainly concern cephalic and pectoral regions. The head becomes shorter and more ventrally oriented during growth. Morphological changes are directly related to the diet shift given that a small mouth ventrally oriented is particularly suited for grazing activities at the adult stage. The pectoral fin is more anteriorely and vertically positioned and its basis is larger in adults than in juveniles. This shape variation had implications for swimming performance, manoeuvrability, turning ability and is related to habitat shift. *Acanthurus triostegus* achieves its main transformation of body shape to an adult-like form at size of 35–40 mm SL.

**Conclusion:**

Most of the shape changes occurred after the reef colonization but before the transition between juvenile habitat (fringing reef) and adult habitat (barrier reef). A large amount of allometric variation was observed after diet shift from zooplankton to benthic algae. Diet shift could act as an environmental factor favouring or inducing morphological changes. On the other hand, the main shape changes have to be achieved before the recruitment to adult populations and start negotiating the biophysical challenges of locomotion and feeding in wave- and current-swept outer reef habitat.

## Background

Ontogenetic shifts in diet and habitat are the norm for demersal marine fishes. The majority of coral reef fishes have stage-structured life histories with two main distinct stages including a pelagic larval stage capable of long-distance dispersal and a demersal stage (usually juveniles and adults) [1]. The transition from the pelagic oceanic environment to benthic reef environment (i.e. settlement phase) represents a key period, during which fish often undergo a change in form (defined as the combination of size and shape) and physiology to a mode suited for the new environment [2]. The settlement phase may be divided into two parts: (1) the fish’s first association with the reef community and (2) a period when fish change to juvenile form [2,3]. As fish grow, their morphology, behaviour and sometimes feeding habit change. Accordingly, some studies have investigated the transition from juvenile to adult habitats (i.e. the recruitment phase) [e.g. [2,4-6]. Four strategies of recruitment according to habitat use were highlighted [5]: (1) an increase in the number of habitats used during the adult stage; (2) a decrease in the number of habitats used by adults compared to recently settled juveniles; (3) the use of different habitat types; and (4) no change in habitat use.

Ecomorphology may assist in our better understanding of the relationships between morphological and ecological changes of an organism during ontogenetic shifts [7,8]. Most ecomorphological investigations have studied the relationships between ontogenetic diet shifts and the changes in oral anatomy [9-11]. Analyses of covariation between feeding habits and external body shape were successfully applied in marine fish inhabiting coastal waters of Mediterranean seas [12-15] whereas, to our knowledge, ecomorphological studies focusing on the post-settlement ontogeny of coral reef fishes are scarce [for exceptions: [16-18]. Allometry refers to the pattern of covariation between size and shape [19] and the study of ontogenetic allometry (i.e. the ontogenetic shape changes within a species when size is used as a proxy of developmental age) has provided insight into regularities of size-required changes in shape for the maintenance of function.

In the present study, the post-settlement development of *Acanthurus triostegus* (L., 1758) was examined. This species is a common coral reef fish in the Indo-Pacific region [20]. In French Polynesia, its larval duration ranges from 44 to 60 days [21]. Typically, juveniles and adults use different habitats [5]. At Moorea Island (Society Archipelago, French Polynesia), *A. triostegus* larvae colonize the reef at night and directly settle on the beach zone [5], which is a shallow sandy area with coral slab. When *A. triostegus* reaches sexual maturity (standard length, SL ≈ 95 mm), they leave the beach community for the barrier reef community where they commonly form large feeding aggregations, which slowly move in the lagoon [5,20]. It is expected that *Acanthurus triostegus* also undergo an ontogenetic diet shift. Indeed, *A. triostegus* is considered as a herbivorous species during its demersal stages [20], as the majority of Acanthuridae. On the other hand, most surgeonfish larvae feed on planktonic preys such as appendicularians [22]. Studies have not yet explored this diet change and have questioned if this shift is rapid or gradual.

Specifically, the present study explores the diet change and the changes in overall body shape during the post-settlement ontogeny of *A. triostegus* (i.e. from settling larvae to adult stage). Two approaches were used to explore potential diet change: stomach content and stable isotope analysis. The combination of these methods has the advantage of compensating for the inaccuracy of each method (see [23] for detailed explanations). Geometric morphometrics was used to study the relationship between body shape variation and changes in feeding habits in a qualitative and quantitative manner. Our approach allowed to highlight a mismatch between morphological changes and ecological shifts in *A. triostegus*. Indeed a large amount of allometric variation was observed after diet shift from zooplankton to benthic algae but the main shape changes were achieved before the recruitment to adult populations living on the outer reef habitat.

## Methods

### Sampling and data collection

All specimens of *Acanthurus triostegus* were collected in the lagoon of Moorea Island (17°30’S, 149°50’W; French Polynesia) in October 2009. The sample represented a complete ontogenetic series from larvae (= larvae settling reef) to adult specimens (n = 117; SL, 22.9–153.8 mm). Larvae (n = 17; SL, 24.5–27.3 mm; Additional file 1) were obtained from nets fixed to the reef crest [24]. Fish captured in crest nets during the night were collected at dawn. Juveniles (n = 87; SL, 22.9–61.0 mm) were caught with a Seine net on the beach zone and adults (n = 13; SL, 104.4–153.8 mm) were speared at dusk just behind the reef crest ( Additional file 1 & Additional file 2). After their capture, the fishes were killed as quickly as possible by an overdose immersion in MS-222 then placed on ice.

**Figure 1:**
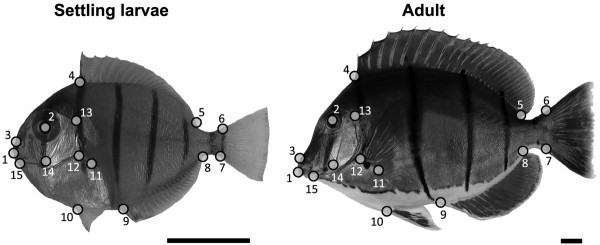
**The homologous landmarks used in the analysis of the fish body shape variation illustrated in settling larvae and adult specimen.** Landmarks (LMs) used in this study defining the overall body shape of * Acanthurus triostegus *. LM (1) mouth tip; LM (2) centre of eye; LM (3) posterior tip of the premaxilla; LMs (4) and (5) anterior and posterior insertion of the dorsal fin; LMs (6) and (7) dorsal and ventral base of the caudal fin; LMs (8) and (9) posterior and anterior insertion of the anal fin; LM (10) insertion of the pelvic fin; LMs (11) and (12) inferior and superior insertion of the pectoral fin; LM (13) most dorso-posterior margin of the opercle; LM (14) point of maximum curvature of the inner edge of the preoperculum; LM (15) insertion of the operculum on lateral profile. Scale bar = 1 cm.

In the laboratory, the SL of each fish was measured to the nearest millimeter with a Vernier caliper. The specimens were photographed in left lateral view with a Canon Powershot S45 camera and the x, y coordinates of 15 homologous landmarks (Figure 1) were digitized using TpsDig [25]. These landmarks were chosen for their capacity to capture overall body shape. Then the entire digestive tract was removed and conserved in 70% alcohol for stomach content analysis. Samples (0.5–2 cm^3^) of lateral muscle tissue of each fish were used for stable isotope analysis. All fish were preserved in 10% neutralized and buffered formalin for 10 days, then transferred to 70% alcohol.

Different potential primary food sources (plankton and algae) were taken from the fish collection site. Meso-zooplankton was trapped using a net with a mesh of 250 μm, towed on the reef at a 2 m depth. Several samples of filamentous algae colonizing the reef were taken and/or brushed from coral slab and rocks: 5 samples were collected on the juvenile habitat and 3 from the adult habitat (Additional file 2).

### Geometric morphometrics

Shape changes were studied using landmark-based geometric morphometric methods [26-28]. An extensive introduction to applications of geometric morphometrics in biology is provided by [29] and [30].

A geometric morphometric analysis involves a series of steps, which are briefly described here. The form of an organism is first captured by the Cartesian coordinates of a configuration of anatomical landmarks (Figure 1). The removal of differences in orientation, position, and size allows pure shape to be analyzed. This was achieved in our study by optimally superimposing landmark configurations using a process called generalized Procrustes analysis (GPA), which is based on a least-squares algorithm [31]. During this superimposition, a consensus configuration (average) of landmarks is calculated and will be used as reference. Centroid size (CS) is a measure of the dispersion of landmarks around their centroid and is computed as the square root of the sum of squared distances of all landmarks from the centroid. Although CS was highly related to SL (linear regression: CS = 1.46 SL (mm) – 0.06; r^2^ = 0.9998, *P* < 0.001), CS was preferred because this is the only measure of size uncorrelated to shape in the absence of allometry [26]. The new Cartesian coordinates obtained after the superimposition are the Procrustes shape coordinates used for statistical comparisons of individuals. The shape differences between landmark configurations of two individuals can be summarized by their Procrustes distance, which is the square root of the sum of squared distances between pairs of corresponding landmarks. Deformation grids using thin-plate spline (TPS) algorithm are commonly used to visualise the patterns of shape variations [26,32,33]. The shape changes modelled by TPS technique can be decomposed into the two components of shape variation, the uniform and non-uniform components. The uniform components (UNI X and UNI Y) express information on global scale shape variation whereas the non-uniform components describe local shape changes at different geometric scales [26]. The first uniform component (UNI X) corresponds to the stretching of a landmark configuration along the x-axis (antero-posterior axis of fish body), whereas the second uniform component (UNI Y) refers to dilatations or compressions along the y-axis (dorso-ventral axis of fish body).

**Figure 2:**
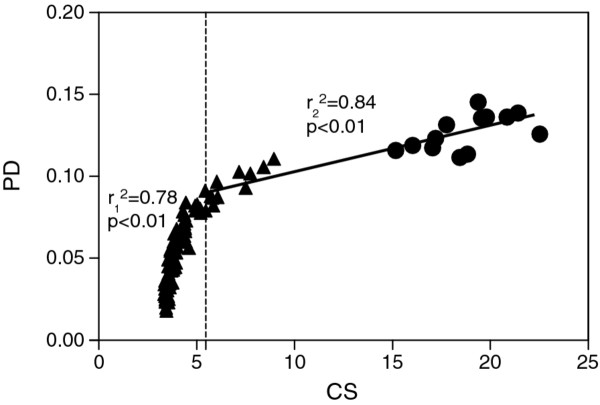
**Plot of Procrustes distance (PD) between individuals and the mean shape of settling larvae on centroid size (CS).** (▲) Juvenile and (●) adult specimens. Dotted line shows the developmental shift for a centroid size value of 5.46 revealed by an adaptive linear regression analysis. The squared coefficient of correlation (r^2^) and *P* value are given for both linear models.

The rate of change in the overall body shape was estimated using the Procrustes distance (PD), which is a proper metric for shape dissimilarity in the Kendall shape space [27]. This distance was used as an univariate measure of shape difference, but needs to be considered as an overall measure of multivariate shape components. The dynamic of shape changes, calculated using Regress6 [34], is visualized by a plot of PD between each specimen and the average shape of the settling larvae in the datasets on their CS. >Since the trajectory describing the dynamic of shape changes was asymptotic (see results, Figure 2), adaptive linear regression splines were computed in R (Version 2.6.10) using the package “Earth” in order to find the transition size allowing the best separation of the dataset.

First, correlation analyses were performed between size (ln-CS) and the X and Y uniform components to test ontogenetic shape changes at global scale. Subsequently, a principal component analysis (PCA) was performed on shape data to identify the major axes of ontogenetic shape changes. PCA was performed using PCAGen6 [35].

### Stomach contents

Stomachs were opened and their contents visually examined using a Wild M10 binocular microscope following the methodology of Wilson & Bellwood [36]. Gut contents were spread over a Petri dish, covering a 15 × 15 square grid placed underneath. For each of 50 randomly marked grid quadrats, the dominant item (by area) was recorded, along with any other material present in the quadrat. The data were condensed into four categories: benthic algae, benthic invertebrates, zooplankton and detritus. According to Randall [20], *A. triostegus* is an herbivorous species at the adult stage. Therefore, we directly calculated the percentage of benthic algae within stomach contents.

### Stable isotopes analysis

Samples of lateral muscle tissue and potential food sources were dehydrated for 24 h at 50°C before being ground into a homogenous powder. After grinding, samples containing carbonates (brushed algae) were placed for 24 h under a glass bell with fuming HCl (37%) (Merck, for analysis quality) in order to eliminate calcareous material. Lipids were not removed from all samples. Carbon and nitrogen gas were analyzed on a V.G. Optima (Micromass) IR-MS coupled to an N-C-S elemental analyzer (Carbo Erba). Routine measurements were precise to within 0.3‰ for both δ^13^C and δ^15^N. Stable isotope ratios were expressed in δ notation according to the following:

(1) $$\delta X=\left[\left(R_{\text{sample}}/R_{\text{standard}}\right)–\;1\right]\times \;1000$$

where *X* is ^13^C or ^15^N and *R* is the corresponding ratio ^13^C/^12^C or ^15^N/^14^N for samples or standards. Carbon and nitrogen ratios are expressed relative to the vPDB (Vienna Peedee Belemnite) standard and to atmospheric nitrogen standard, respectively. Reference materials were IAEA-N1 (δ^15^N = +0.4 ± 0.2‰) and IAEA CH-6 (sucrose) (δ^13^C = −10.4 ± 0.2‰).

## Results

### Geometric morphometrics

The dynamic of shape changes during ontogeny, expressed as the relationship between PD and CS, is on Figure 2. The trajectory displays an asymptotic curve. The rate of shape changes is high for small individuals and decreases during growth. An adaptive linear regression analysis highlighted a developmental shift for a centroid size value of 5.46, corresponding to 37.8 mm of SL. Consequently, the plateau is reached in the size range of juveniles, revealing that the habitat shift undergone by *A. triostegus* during the recruitment (i.e. the change from the beach zone to the barrier reef) occurs when all shape changes are almost completed.

**Figure 3:**
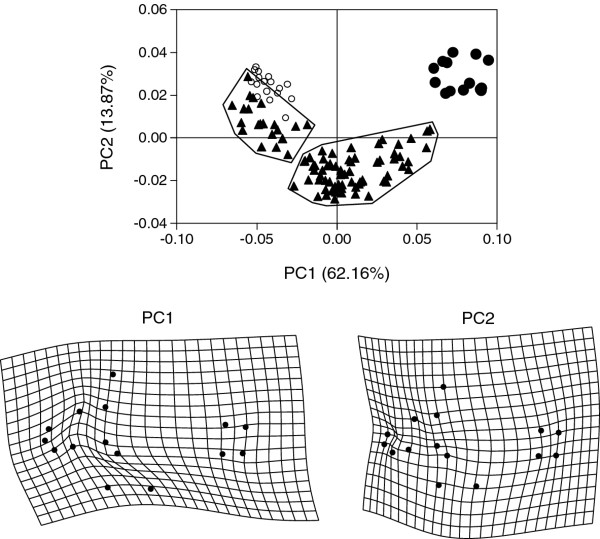
**Principal components analysis illustrating the ontogenetic trajectory of** *** Acanthurus triostegus *** **in the shape space.** Plots of the first two PCs; percentage of shape variance summarized by each PC is given in parentheses. (○) Larvae at settlement, (▲) Juvenile and (●) adult specimens. The polygons illustrate the visual discrimination of two groups in juveniles. TPS-grids illustrate shape changes associated with the first two principal components. PC1-grid and PC2-grid span across a total of 0.1 axis unit (−0.05 to 0.05) and 0.06 axis unit (−0.03 to 0.03), respectively. The shape differences have been exaggerated for better visualization (PC1: x1.5; PC2: x2.5).

Correlation analysis between UNI X and ln-CS highlighted a global stretching of the body shape along the antero-posterior axis with *A. triostegus* growth (r = 0.87, *P* < 0.001). On the other hand, UNI Y was poorly correlated to ln-CS (r = 0.22, *P* = 0.02) suggesting no global compression of the body shape along the dorso-ventral axis during growth. The ontogenetic trajectory of *A. triostegus* is curvilinear in the shape space defined by the first two principal components. Without a priori determination of groups, the juveniles could be divided into two groups: one showing relatively similar shape than settling larvae (a) and the others (b, Figure 3). Both juvenile groups showed significant difference in mean body size (Mann Whitney test *P* < 0.001). PC1 explains 62.2% of the total shape variance. TPS deformation grids, used to interpret shape changes associated with PC1, show that adults have (1) a more elongate body, (2) a shorter head (LMs 1, 4, 13–15), (3) a longer dorsal fin (LMs 4 and 5), (4) a mouth more ventrally oriented (LMs 1, 3, 15), (5) a less high ventral region (LMs 9–12) and (6) a larger base for the pectoral fins which are also more anteriorely and vertically positioned (LMs 11 and 12) (Figure 3). PC2 explains 13.9% of the variance and highlights a biphasic development since PC2 scores decrease during a first part of the ontogeny while an increase of PC2 scores is observed during a second phase. PC2 mainly express variations of the frontal region (LMs 3 and 4), the mouth (LMs 1, 3, 15) and the ventral region (LMs 9–12) (Figure 3).

### Stomach contents

**Figure 4:**
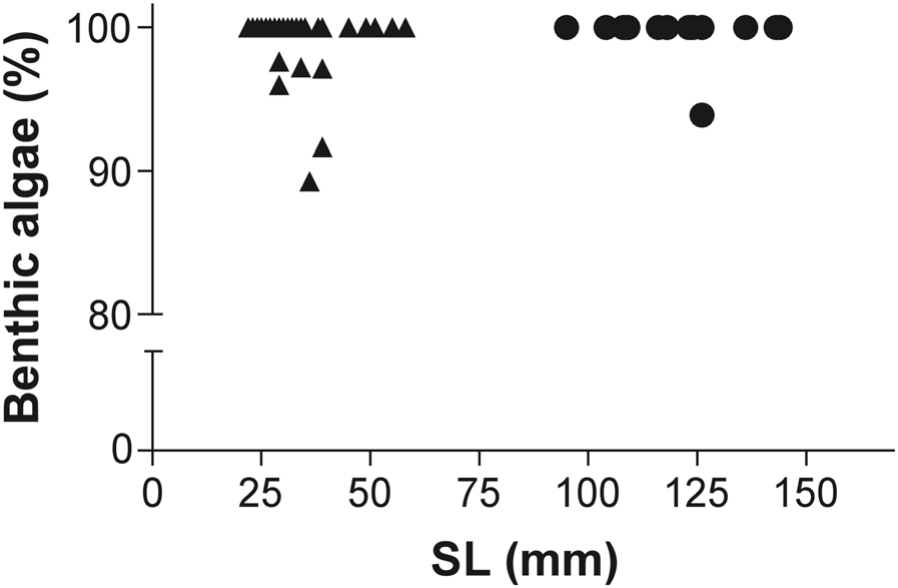
**Percentage of benthic algae presents in the stomach content of** *** Acanthurus triostegus. *** Scatterplot illustrating the relationship between the percentages of benthic filamentous algae found in the stomach contents and fish size (SL, mm). (▲) Juvenile and (●) adult specimens. Settling larvae and some juveniles are lacking because they had empty stomach.

The stomach of all larvae captured during settlement events and five juveniles was empty. Juveniles and adults mainly fed on benthic algae (> 75% of the stomach content; Figure 4). The change from planktivory to herbivory seems to be instantaneous over post-settlement ontogeny. The type of algae found in stomach contents was mainly green filamentous algae. Sediment is the second most recurrent item found in stomach of *A. triostegus*.

### Stable isotopes

**Figure 5:**
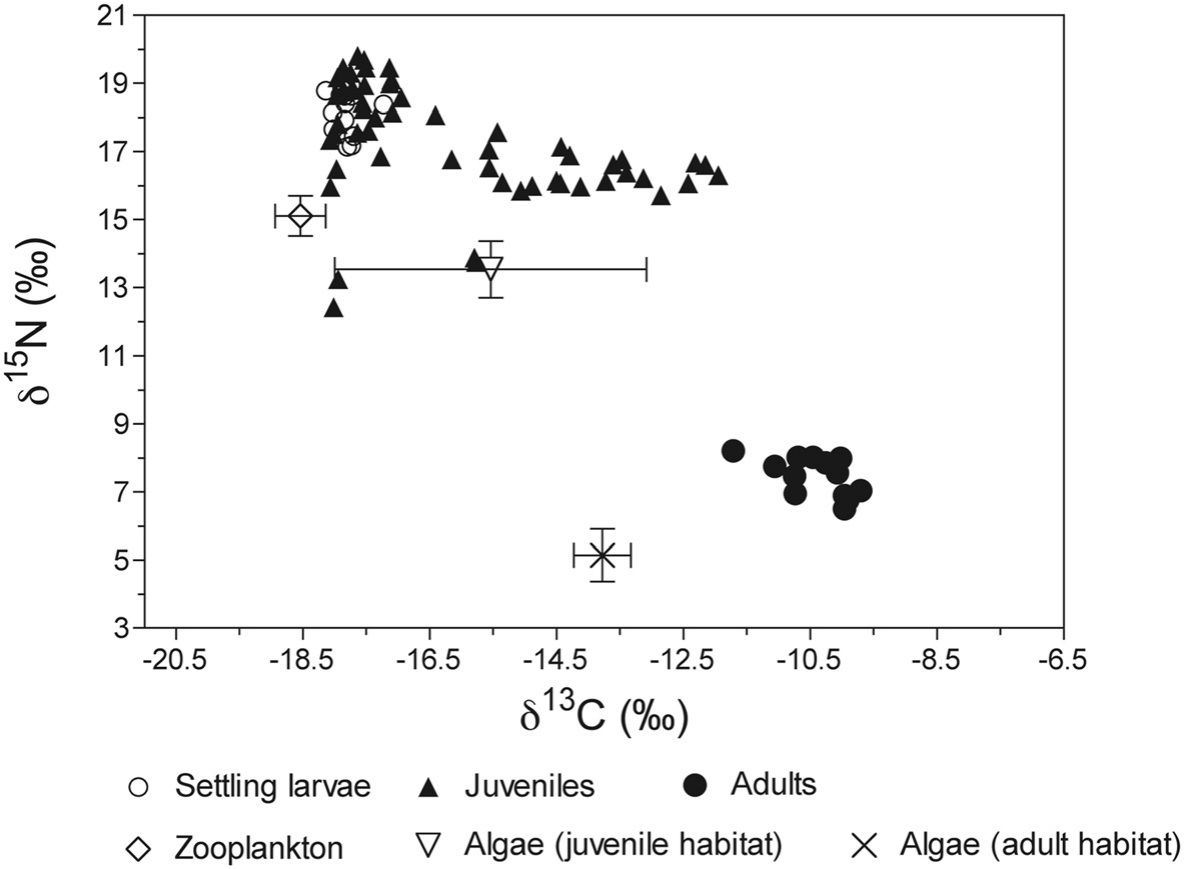
**Isotopic map of sample populations of** *** Acanthurus triostegus *** **and their food sources.** Mean (± SD) d15N and d13C ratios of food items (zooplankton and algae from both juvenile and adult habits) collected from the lagoon and isotope values of all * Acanthurus triostegus * (settling larvae, juveniles, adults).

Zooplankton had lower δ^13^C and higher δ^15^N than algae (Figure 5). Isotopic values in algae from the juvenile habitat ranged from −18.0 to −12.2‰ for δ^13^C and from 12.2 to 14.5‰ for δ^15^N. Benthic algae from the adult habitat had significant lower δ^15^N than algae from the juvenile habitat (Mann Whitney test, *P* = 0.036).

There were significant differences between the delta values among settling larvae, juveniles and adult specimens of *A. triostegus* (Kruskal-Wallis test: δ^13^C, H_2_ = 43.96, *P* < 0.001; δ^15^N, H_2_ = 37.93, *P* < 0.001). Except settling larvae and juveniles showing no difference along the δ^15^N axis (Dunn’s Multiple comparison test, *P* > 0.05), all other paired comparisons among groups revealed significant differences (*P* < 0.001). Adults showed the least negative δ^13^C values and settling larvae had the most negative δ^13^C values. Juveniles presented the greater variation of δ^13^C values. A group of juveniles had very similar δ^13^C and δ^15^N values than settling larvae when other juveniles form a group showing a large range of δ^13^C values. Interestingly, the δ^13^C variation observed in juveniles is significantly related to fish size (δ^13^C Vs SL: r^2^ = 0.26; *P* < 0.001) and shape (δ^13^C Vs PC1 r^2^ = 0.38; *P* < 0.001). Moreover these linear models fitted better when the five largest juveniles are excluded from the regression analysis (δ^13^C Vs SL: r^2^ = 0.62; *P* < 0.001; δ^13^C Vs PC1: r^2^ = 0.47; *P* < 0.001) (Additional file 3). Settling larvae and adults showed the most positive and the lowest δ^15^N values, respectively.

## Discussion

**Figure 6:**
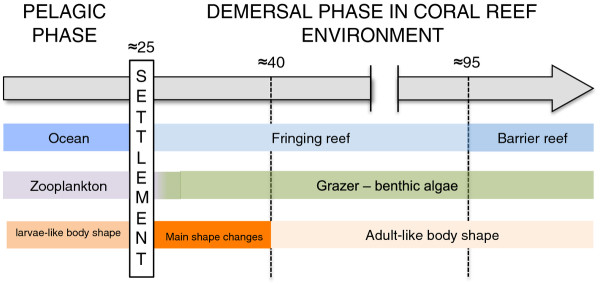
**Schematic representation of the ecological and shape changes observed during the ontogeny of** *** Acanthurus triostegus. *** See the text for details. The grey arrow represents the ontogenetic scale. The numbers above the scale refer to the approximate fish size (standard length in mm) when the changes occur. Variations in habitat, diet and morphology are illustrated by a gradient of blue, green and orange, respectively.

After reef settlement, the ontogeny of *Acanthurus triostegus* is highly allometric. The main shape changes occurred before the transition between juvenile and adult habitat. However, *A. triostegus* changed to an herbivorous diet before most ontogenetic shape changes occurred. Although a global lengthening of the whole body was observed during growth, the main shape variation concerned the anterior half part of the body, with the pectoral girdle becoming more anteriorely positioned and the mouth becoming more ventrally oriented during growth. Figure 6 summarizes the ontogenetic shifts observed by *A. triostegus* during its post-settlement growth.

For demersal coral reef fishes, there is currently little information connecting morphological changes with ecological changes [e.g. [2]. On the other hand, this kind of data is growing for demersal reef fishes from temperate regions [e.g. [12-15,37]. Similar to sparids [12,13,15] and coral reef damselfishes [16], the dynamic of shape changes in *A. triostegus* was asymptotic, reflecting a two-step curve (Figure 2). *Acanthurus triostegus* achieved their main transformation of body shape to an adult-like form at a size of 35–40 mm SL. Our findings that the mean size of settlement-stage of *A. triostegus* is 25 mm SL [present study, [20] and growth rate is a mean of 20 mm SL per month [20] reveal that the main changes in body shape occur during the first month in the coral reef environment. This stage, corresponding to a shift of allometric pattern, does not coincide with the shift of habitat (Additional file 2, Figure 6). Indeed, the main changes of body shape are completed before the transition from the fringing reef (juvenile habitat) to the reef crest of the barrier reef (adult habitat) [5], suggesting that fish should acquire a specific body shape to be competitive compared to adults already installed in the reef crest community (Figure 6).

Metamorphosis in fish requires radical changes in external and internal morphology, and is often accompanied by ecological or habitat changes [38]. Generally, metamorphosis is thought to be rapid, usually occurring within a few hours of settlement [39]. Information about metamorphosis in *A. triostegus* is currently growing. McCormick [21] highlighted changes in pigmentation and body shape variation in *A. triostegus* during the first ten days after reef settlement. The main shape changes are a reduction of the second dorsal spine and a reduction of the body depth [21]. After 5 days post-settlement, the mouth starts to move from a terminal to a ventral position [21]. More recently, Frédérich *et al.*[40] highlighted an unexpected skeletal change during its metamorphosis. They observed a rapid replacement of dermal plates to small scales during the 6–9 days post-settlement. Concerning ecological changes, it is already known that juveniles and adults use different habitat [5]. At Moorea Island, the juveniles of *A. triostegus* grow on beach zones characterized by a shallow sandy area with coral slab whereas adults inhabit the lagoon to the shoreward side of the barrier reef crest. Our isotopic data clearly demonstrate the ontogenetic diet shift between settling larvae and adults of *A. triostegus* (Figure 5). Indeed, larvae and adults had δ^13^C and δ^15^N values that matched the isotopic signatures of zooplankton and benthic algae, respectively. The size related variation of δ^13^C observed in juveniles should reflect tissue turnover [41]. Knowing that the small juveniles having similar δ^13^C and δ^15^N values than settling larvae already graze filamentous algae revealed the tissue turnover is not instantaneous and some time is needed to reach isotopic equilibrium [42]. On the other hand, the lower values of δ^15^N observed in adults than in juveniles and their respective algal food sources have to be related to habitat shift and/or the different nutrient sources for benthic algae between juvenile and adult habitats [43]. Indeed, juvenile habitat is close to the beach (our sample site was near a hotel) and an influence of anthropogenic nitrogen inputs on δ^15^N seems possible [44]. During their pelagic larval phase, *A. triostegus* feeds on appendicularians and larval polychaetes [20,22]. Unfortunately, the stomach of all larvae captured during the settlement event was empty. However, benthic algae represented more than 75% of the gut content in all juveniles caught on the beach zone. Knowing that the smallest juveniles caught in the lagoon are similar size to the settling larvae caught using the crest net, suggests the diet change from a planktivorous regime to an herbivorous one should be rapid. Surprisingly, our study reveals that a large amount of allometric shape changes remain to be done after the diet shift has occurred and before the recruitment in adult populations (Figure 6). The quick diet shift after settlement could act as an environmental factor favouring or inducing the main morphological changes at least at the level of the cephalic region. The external morphology of the smallest *A. triostegus* juveniles would be sufficient for efficient grazing during the first post-settlement days, but the rapid transition to an algivorous regime could also be possible due to physiological adaptations (e.g. new set of digestive enzymes) or morphological adaptations of the digestive tract. In any case, we observed a rapid lengthening of the intestine in *A. triostegus* juveniles during the first few days post-settlement [Frédérich, pers. obs.]. The timing of metamorphosis in coral reef fishes varies according to fish groups and is directly linked to species-specific ecological transition periods [2,3]. If the concept of metamorphosis includes the gradual transformations during the juvenile phase, we have demonstrated that *A. triostegus* achieve their metamorphosis to an adult-like form at a size of 35–40 mm SL (i.e. approximately one month after settlement to the reef).

The allometric shape changes mainly concern the cephalic region and the pectoral region. Conversely, the caudal region did not vary over ontogeny. During growth, the head becomes shorter and more ventrally oriented. The variation of mouth orientation is mainly due to a lengthening of the frontal region and may be correlated with a heightening of the cheeks. These shape changes are directly related to the diet shift. Indeed, a small mouth ventrally oriented is particularly suited for grazing activities at the adult stage [45,46]. The pectoral fin is more anteriorely and vertically positioned and its basis is larger in adults than in juveniles. This morphological variation of the pectoral region is directly related to ontogenetic habitat shift as suggested by various ecomorphological studies about swimming in coral reef fishes. Juveniles of *A. triostegus* live a calm inshore environment when adults swim in wave- and current-swept outer reef habitat. Fulton [47] showed the adults of *A. triostegus* use essentially their pectoral fins for swimming (Labriform swimming mode sensu [48] and [49]) and is one the most powerful swimmers in wave-swept reef crest habitat. Consequently, a larger basis of their pectoral fins observed in adults suggests the development of powerful muscles needed for high swimming speed performance [47]. A more anteriorely positioned pectoral fin in adults is linked to a better ability to break forward movement and thus fine-tune locomotion in quarters of complex substrata [50]. Finally, a more vertically oriented pectoral fin allows a better manoeuvrability and turning ability in adults [51], another benefit in fast waters and structurally complex environment.

## Conclusion

Overall, this study is one of the first to (1) detail quantitatively and qualitatively the complete ontogenetic shape changes of a surgeonfish occurring after reef settlement and to (2) highlight a mismatch between morphological changes and ecological shifts in coral reef fishes. Indeed, a large amount of allometric shape changes remains to be done after the diet shift when most of the shape changes are completed before the second habitat shift from beach zone to barrier reef. Our morphological study shows that cephalic and pectoral regions undergo a large amount of allometric shape changes through post-settlement ontogeny. The number of works focusing on the various modes of post-settlement transition in coral reef fishes is growing [5,52] but studies investigating the morphological, physiological and ethological adaptations associated to these transition periods are also needed. The magnitude and the disturbance of such adaptations can directly impact levels of mortality occurring at each stage of ontogenetic development after reef settlement.

## Competing interests

The authors declare that they have no competing interests.

## Authors’ contributions

BF and DL conceived of the study and both collected specimens in the field. BF carried out dissections, morphometric analyses and analysed the data. BF, OC and GL carried out stomach content and stable isotope analysis. BF wrote the paper with input from DL, OC and GL. All authors read and approved the final manuscript.

## Supplementary Material

Additional file 1 **Box plot illustrating body size distribution within settling larvae, juveniles and adults.** Median, percentiles 25–75%, maximum and minimum values are illustrated. Click here for file

Additional file 2 **Sampling locations in Moorea Island (Society Archipelago, French Polynesia)**. White cross and star refer to sampling area for juveniles and adults, respectively. Click here for file

Additional file 3 **Relationships between (A–B) δ**^**13**^**C values and fish size (CS), and (C–D) δ**^**13**^**C values and shape (PC1) in** ***Acanthurus triostegus*** **living in the juvenile habitat** ***.*** Regressions were produced with all juveniles (A & C) and excluding the four largest juveniles (B & D). Equation of regression models: (A) δ^13^C = 0.7CS-19.4; (B) δ^13^C = 1.8CS-23.8; (C) δ^13^C = 34.3PC1–16.0; (D) δ^13^C = 40.5PC1–15.8. Click here for file
