# Supplementary figures and images for: Mismatch between shape changes and ecological shifts during the post-settlement growth of the surgeonfish, Acanthurus triostegus
Source: Front Zool. 2012 Apr 25;9:8. doi: 10.1186/1742-9994-9-8 (PMC3495409; doi:10.1186/1742-9994-9-8)

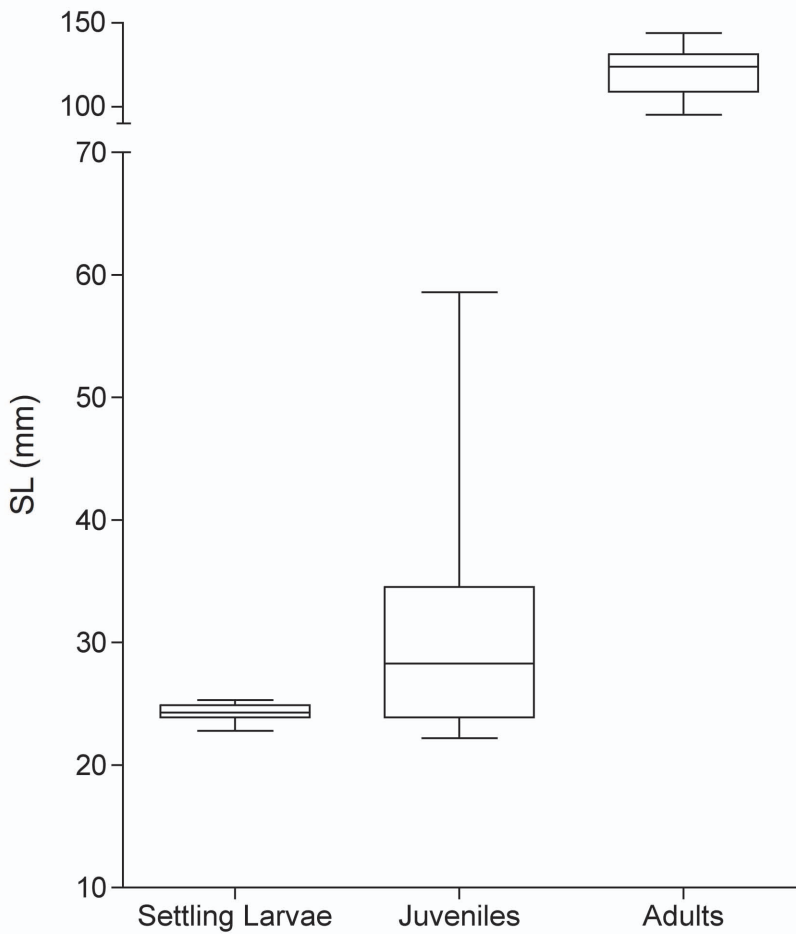

Supplement: Additional file 1 — Box plot illustrating body size distribution within settling larvae, juveniles and adults. Median, percentiles 25–75%, maximum and minimum values are illustrated. [file 1742-9994-9-8-S1.pdf]

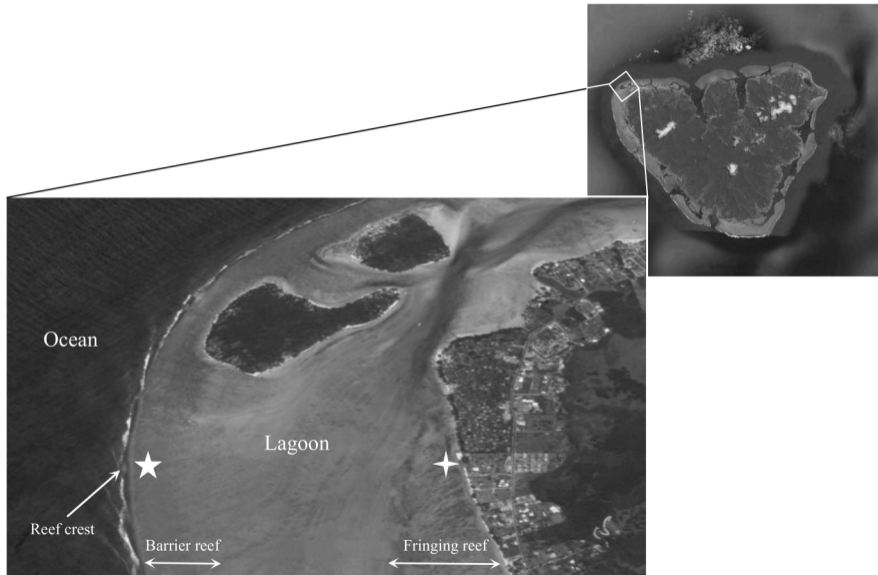

Supplement: Additional file 2 — Sampling locations in Moorea Island (Society Archipelago, French Polynesia). White cross and star refer to sampling area for juveniles and adults, respectively. [file 1742-9994-9-8-S2.pdf]

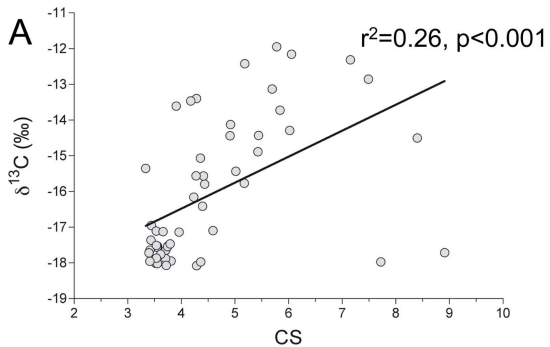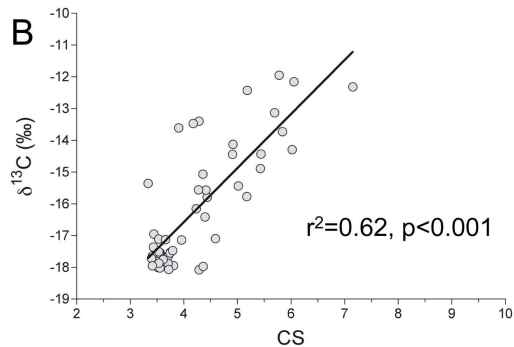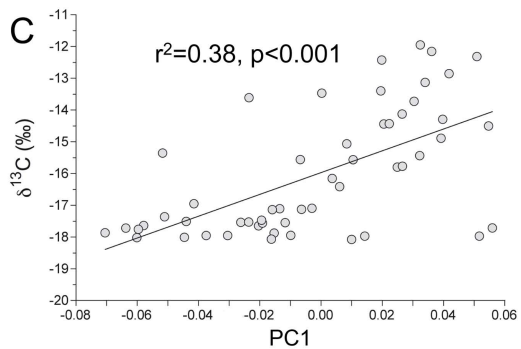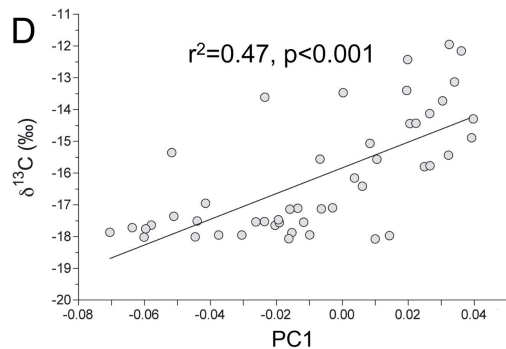

Supplement: Additional file 3 — Relationships between (A–B) δ 13 C values and fish size (CS), and (C–D) δ 13 C values and shape (PC1) in Acanthurus triostegus living in the juvenile habitat . Regressions were produced with all juveniles (A & C) and excluding the four largest juveniles (B & D). Equation of regression models: (A) δ13C = 0.7CS-19.4; (B) δ13C = 1.8CS-23.8; (C) δ13C = 34.3PC1–16.0; (D) δ13C = 40.5PC1–15.8. [file 1742-9994-9-8-S3.pdf]
